# AMPK/ SIRT1 signaling pathway activation acts on PGC-1α/ PPARγ to alleviate sepsis-acquired weakness

**DOI:** 10.1038/s41420-026-03212-w

**Published:** 2026-06-23

**Authors:** Lu Li, Lingyan Shi, Meirong Liu, Qi Fang

**Affiliations:** 1 Department of Neurology, The First Affiliated Hospital of Soochow University, Suzhou, Jiangsu 215006 China; 2 Department of Electrophysiology, Weihai Municipal Hospital, Cheeloo College of Medicine, Shandong University, Weihai, Shandong 264200 China; 3 Department of Critical Care Medicine,Weihai Municipal Hospital, Cheeloo College of Medicine, Shandong University, Weihai, Shandong 264200 China

**Keywords:** Cell biology, Molecular biology

## Abstract

Intensive care unit-acquired weakness (ICU-AW) represents a critical complication that severely impairs the prognostic outcomes of patients with sepsis. This study aimed to elucidate whether the activation of the adenosine 5’-monophosphate-activated protein kinase (AMPK)/silent information regulator 1 (SIRT1) signaling pathway ameliorates sepsis-acquired weakness (SAW) via modulating the peroxisome proliferator-activated receptor γ coactivator-1α (PGC-1α)/peroxisome proliferator-activated receptor γ (PPARγ) axis. A PGC-1α knockout mouse model was established using the cecal ligation and puncture (CLP) method to induce sepsis and subsequent SAW. Multiple histological analyses were performed to evaluate tissue pathological lesions, mitochondrial morphological and structural abnormalities, myelin injury, and skeletal muscle alterations. ELISA was utilized to quantify the expression levels of pro-inflammatory factors and neurotransmitters. Western blotting and RT-qPCR were further employed to detect the expression of mitochondrial functional proteins and downstream molecules associated with the AMPK/SIRT1 signaling pathway. Successful construction of the PGC-1α knockout CLP mouse model was confirmed via systematic verification. Functional experiments validated that AMPK/SIRT1 pathway activation exerts protective effects against SAW in CLP mice through targeting the PGC-1α/PPARγ signaling cascade. Mechanistically, AMPK/SIRT1 activation further modulates the PGC-1α/PPARγ pathway, thereby alleviating the progression of SAW. Collectively, targeted regulation of PGC-1α may serve as a promising and actionable therapeutic strategy for the clinical prevention and treatment of ICU-AW in septic patients.

**Figure:**
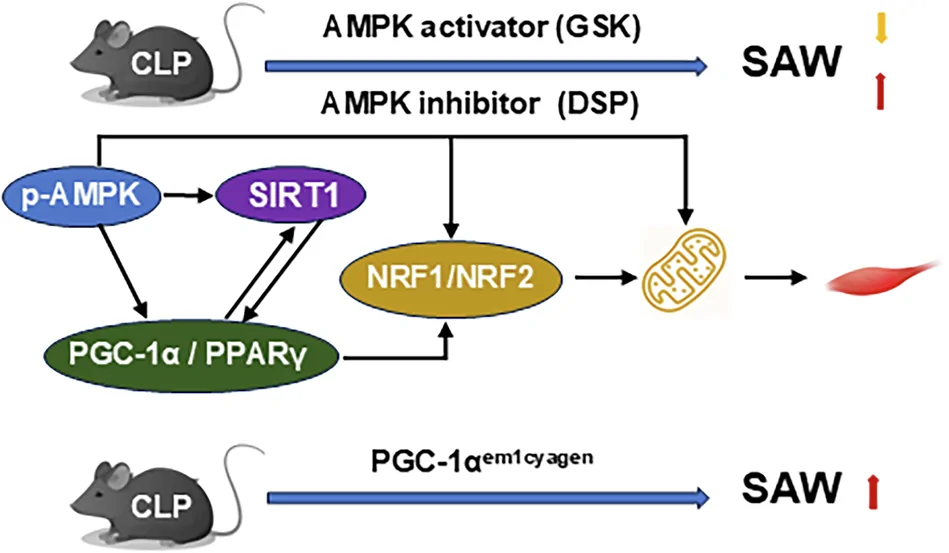

## Background

Sepsis, a common life-threatening disease in the intensive care unit (ICU), is characterized by systemic excessive inflammatory response and multiple organ dysfunction [1]. With persistently high morbidity and mortality, it imposes a substantial burden on the global healthcare system [2]. During the recovery phase of sepsis patients, ICU-acquired weakness (ICU-AW) has emerged as one of the most prevalent complications, clinically manifesting as generalized skeletal muscle weakness, atrophy, and motor dysfunction [3, 4]. This condition significantly compromises patients’ success rate of weaning from mechanical ventilation, rehabilitation progress, and long-term quality of life [5]. As a key subtype of ICU-AW, the pathogenesis of sepsis-acquired weakness (SAW) arises from the complex interplay of four core pathological cascade reactions: inflammation-driven injury, energy failure, inadequate tissue perfusion, and metabolic imbalance [6, 7]. Specifically, its pathogenic mechanisms center on the multifaceted damage inflicted by sepsis-induced systemic pathological conditions on skeletal muscle, mitochondria, and the neuromuscular transmission system [8]. Although existing studies have confirmed that inflammation-mediated muscle cell injury and mitochondrial dysfunction are critical links in the development of SAW, research on targeted interventions for the underlying molecular pathways remains inadequate.

Peroxisome proliferator-activated receptor γ coactivator l alpha (PGC-1α) is a crucial regulator of mitochondrial biogenesis and quality control mechanisms such as fission, fusion, and dynamics, including fission and fusion [9]. These physiological processes regulate muscular contraction and relieve symptoms of SAW, such as muscle weakness resulting from impaired NMJ propagation. PGC-1α is a protein that enhances the activity of peroxisome proliferator activated receptor Gamma (PPARγ). PPARγ synergizes with PGC-1α to maintain the functional homeostasis of skeletal muscle mitochondria by regulating mitochondrial biogenesis, lipid metabolism, and oxidative stress balance, thereby facilitating metabolic recovery and cellular remodeling. Extracts of *Ficus carica L*. can inhibit inflammation and alleviate muscle atrophy by upregulating PPARα expression and suppressing NF-κB activation [10, 11].

During the process of muscle adaptation, the adenosine 5‘-monophosphate (AMP)-activated protein kinase (AMPK) enhances the production of adenosine 5’-triphosphate (ATP) by regulating many genes and proteins, such as PGC-1α, PPAR, and mitochondria-associated proteins [12]. Such signaling cascades play a pivotal role in boosting muscle metabolic activity. Silent information regulator 1 (SIRT1) is an enzyme that relies on nicotinamide adenine dinucleotide (NAD) to remove acetyl groups from proteins. It interacts with PGC-1α through alterations that occur after the transcription of genetic information, in order to regulate the creation of new mitochondria [13]. Research has indicated that the stimulation of AMPK/SIRT1/PGC-1α-dependent mitochondrial biogenesis in skeletal muscle can potentially safeguard skeletal muscle against muscular dysfunction, insulin resistance, and cognitive deficiencies that may occur naturally during life [14–16]. Nevertheless, there is a lack of research on the role of AMPK/SIRT1 in neuromuscular dysfunction caused by sepsis. The main objective of our work is to investigate whether the AMPK/SIRT1 pathway can enhance sepsis-induced neuromuscular dysfunction by modulating mitochondrial activity through its impact on PGC-1α/PPARγ.

## Results

### Activation of AMPK/SIRT1 signaling pathway improves SAW in CLP mice

**Fig. 1:**
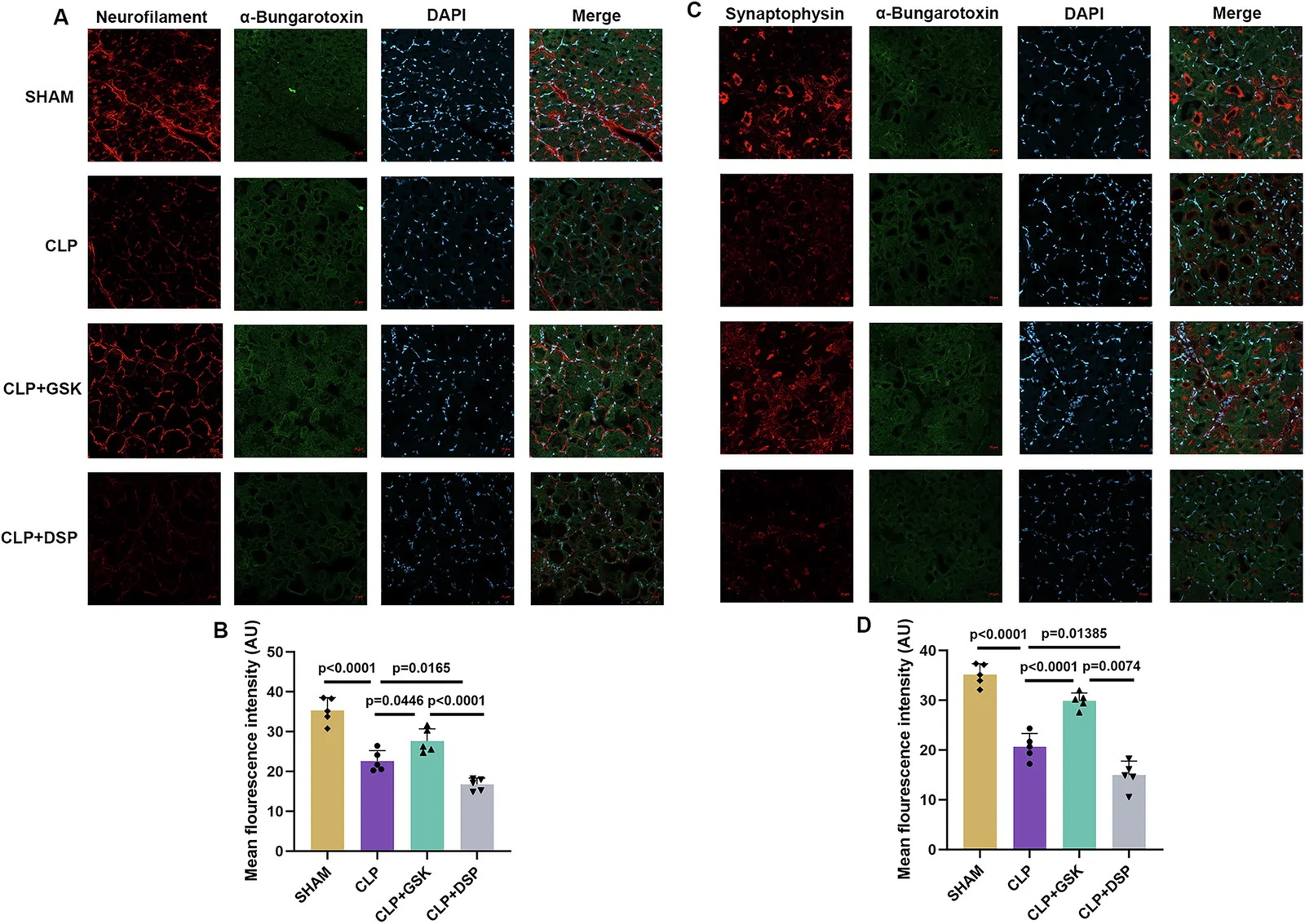
AMPK/SIRT1 signaling pathway can modulate neuromuscular dysfunction in septic mice. **A**, **B** Neurofilament/α-Bungarotoxin immunofluorescence plots and data analysis. **C**, **D** Synaptophysin/α-Bungarotoxin immunofluorescence plots and data analysis. Red, Neurofilament/ Synaptophysin; Green, α-Bungarotoxin; Blue, DAPI. SHAM, Sham operation on mice; CLP, Mouse models of sepsis; CLP + GSK, AMPK activator GSK621-injected mice with sepsis; CLP + DSP, AMPK inhibitor dorsomorphin-injected mice with sepsis. Mean ± SD. *n* = 5. (scale bar, 20 μm).

Neurofilament (NF) is a prominent protein found in the cytoskeleton of neuronal axons. It is primarily present at synaptic locations in neurons to uphold the stability of axon structure and facilitate proper neuronal signaling [17]. Synaptophysin (SYP) is a protein that is exclusive to neurons and has a role in the packaging and transportation of neurotransmitters [18]. To investigate the regulatory significance of the AMPK/SIRT1 signaling pathway in sepsis-acquired weakness, we created a CLP mouse model and delivered treatments using an AMPK activator (GSK621) and an AMPK inhibitor (DSP). We employed the immunofluorescence double labeling technique to identify the simultaneous presence of NF/SYP and α-Bungarotoxin (labeling postsynaptic acetylcholine receptors) in order to assess the muscular nerve function of mice (Fig. 1). The findings demonstrated a sequential decline in the expression level of NF/SYP in the muscles of SHAM mice, CLP mice, and CLP + DSP mice. However, treatment with GSK621 significantly reversed the CLP-induced reduction in NF/SYP expression (CLP + GSK vs. CLP, Fig. 1A–D).

**Fig. 2:**
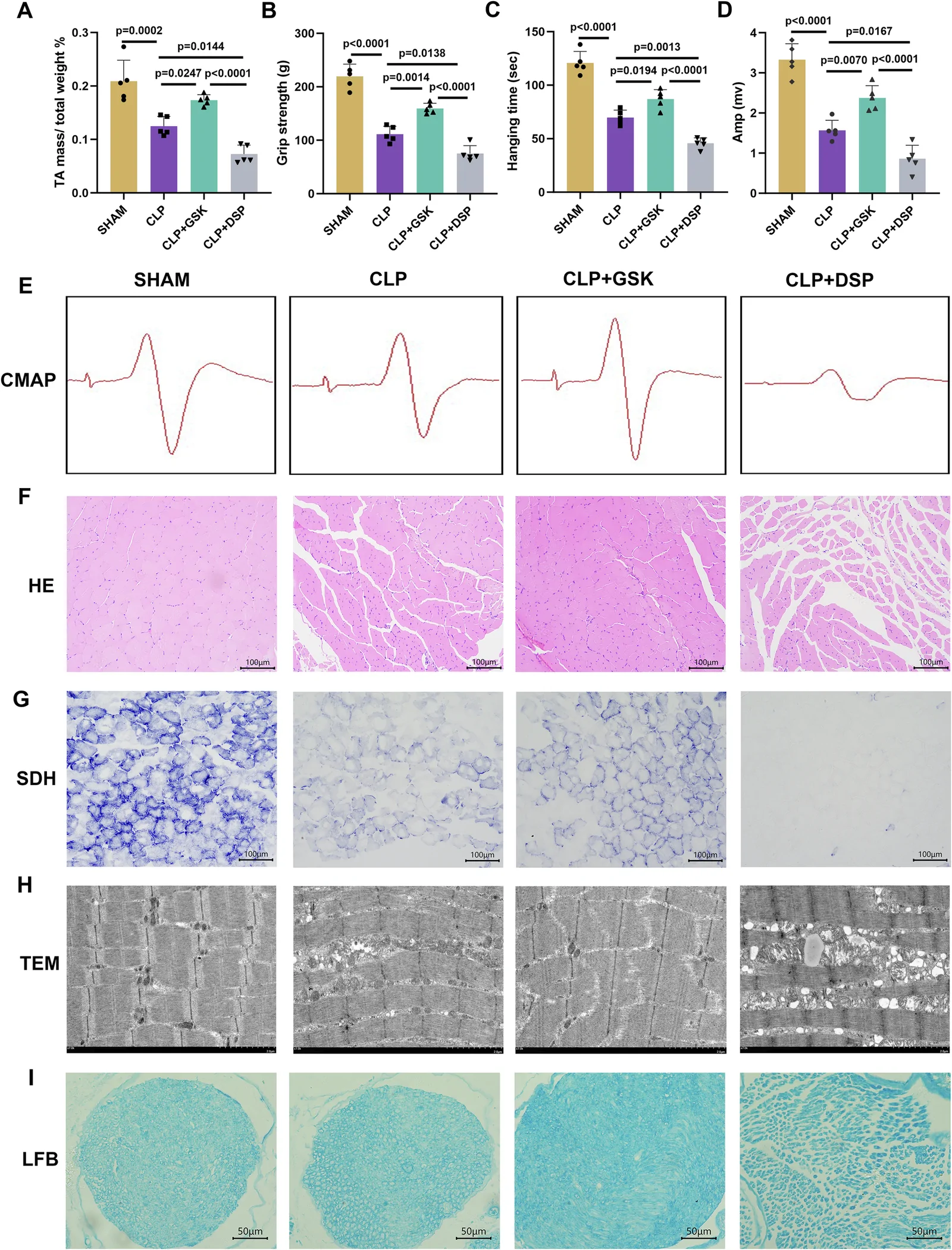
Modulation of the AMPK/SIRT1 signaling pathway alleviates SAW in CLP mice. **A** The TA mass/total weight of mice. **B** The grip strength of mice. **C** The hanging time of mice. **D**, **E** CMAP for the detection of muscle weakness status in mice and its quantitative analysis. **F** HE staining of mouse muscle tissue. **G** SDH staining of mouse muscle tissue. **H** Mitochondrial ultrastructure observed by TEM of mouse muscle tissue. **I** LFB staining of mouse muscle tissue. TA tibialis anterior muscle, CMAP compound muscle action potential, HE hematoxylin-eosin, SDH, succinate dehydrogenase, LFB luxol fast blue, TEM transmission electron microscope; Mean ± SD. *n* = 5. (scale bar, HE/SDH, 100 μm; LFB, 50 μm; TEM, 2 μm).

To study the pathological and behavioral changes of muscle atrophy caused by CLP, we evaluated the TA muscle content, grip strength, and suspension time of mice in each treatment group. The results showed that the TA muscle content (Fig. 2A), grip strength (Fig. 2B), and hanging time (Fig. 2C) of CLP mice were significantly lower than those of the SHAM group (*P* < 0.05). After adding GSK621 and DSP treatment, obvious changes occurred. Behavioral experiments have shown that AMPK is involved in the muscle atrophy process triggered by CLP. The CMAP results in mice showed that CLP mice had a higher degree of muscle weakness. In addition, the intervention of DSP aggravated muscle weakness in CLP mice, whereas GSK621 repaired the symptoms of muscle weakness in mice (Fig. 2D, E). Subsequently, we conducted histological verification to confirm that the regulation of the AMPK/SIRT1 signaling pathway could effectively reduce SAW in CLP mice. The HE staining results revealed that wild-type mice exhibited muscle fibers that were consistently arranged, myocytes with a typical shape. The myocytes of CLP mice exhibited cellular swelling and disarrayed nuclear organization. The CLP + DSP mice exhibited the most pronounced pathological alterations, characterized by larger regions displaying degenerative changes. The intervention by GSK621 has the potential to greatly improve the muscle pathology in mice (Fig. 2F).

SDH serves as a crucial link between oxidative phosphorylation and electron transport. It supplies electrons to mitochondria and the respiratory chain, which are essential for meeting the cellular requirement for oxygen and energy generation [19]. The findings of our SDH staining demonstrated a gradual decrease in SDH content in the muscles of SHAM mice, CLP mice, and CLP + DSP mice. However, the administration of GSK621 considerably enhanced the SDH content (Fig. 2G), TEM analysis revealed a high abundance of perinuclear mitochondria in the SHAM mice. These mitochondria exhibited distinct and well-defined structures, together with orderly ordered muscle filaments and undamaged muscle membranes. The mitochondria of CLP mice exhibited swelling and degeneration, accompanied by alterations in their cristae structure, such as the mitochondrial cristae displaying fragmentation, dissolution, and the formation of empty spaces. The mitochondrial membrane of CLP + DSP mice exhibited a deficiency, which was further exacerbated. The intervention of GSK621 considerably improved the mitochondrial shape and structure of CLP mice (Fig. 2H). The LFB staining results indicated that the nerve tissue of CLP mice suffered damage. Additionally, the intervention of DSP worsened the nerve damage in CLP mice, while GSK621 repaired the nerve injury (Fig. 2I).

**Fig. 3:**
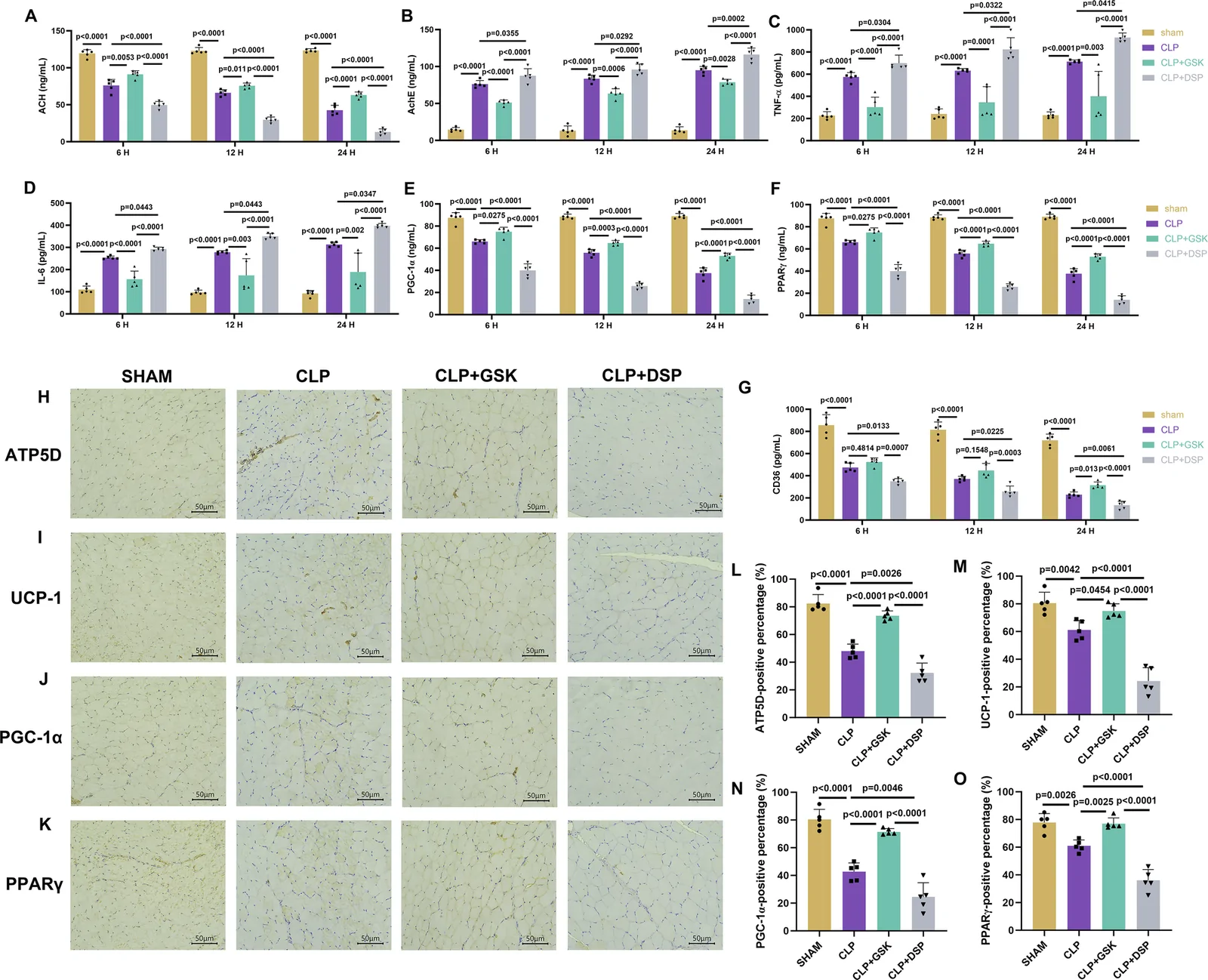
Modulation of the AMPK/SIRT1 signaling pathway alleviates SAW in CLP mice. **A**–**G** ELISA for determination of cytokine levels in mouse serum at different time points (6/12/24 h) after modeling. **H**–**K** Immunohistochemical staining for ATP5D/ UCP-1/ PGC-1α/ PPARγ. **L**–**O** Quantitative analysis of immunohistochemical staining of mouse muscle. ACH acetylcholine, AchE acetylcholinesterase, TNF-α tumor necrosis factor alpha, IL-6 interleukin 6, CD36 cluster of differentiation 36, ATP5D ATP Synthase 5 Delta, UCP-1 uncoupling protein 1, PGC-1α peroxisome proliferator-activated receptor γ coactivator lalpha, PPARγ peroxisome prolilerators-activated receptor γ; Mean ± SD. *n* = 5. (scale bar, 50 μm).

**Fig. 4:**
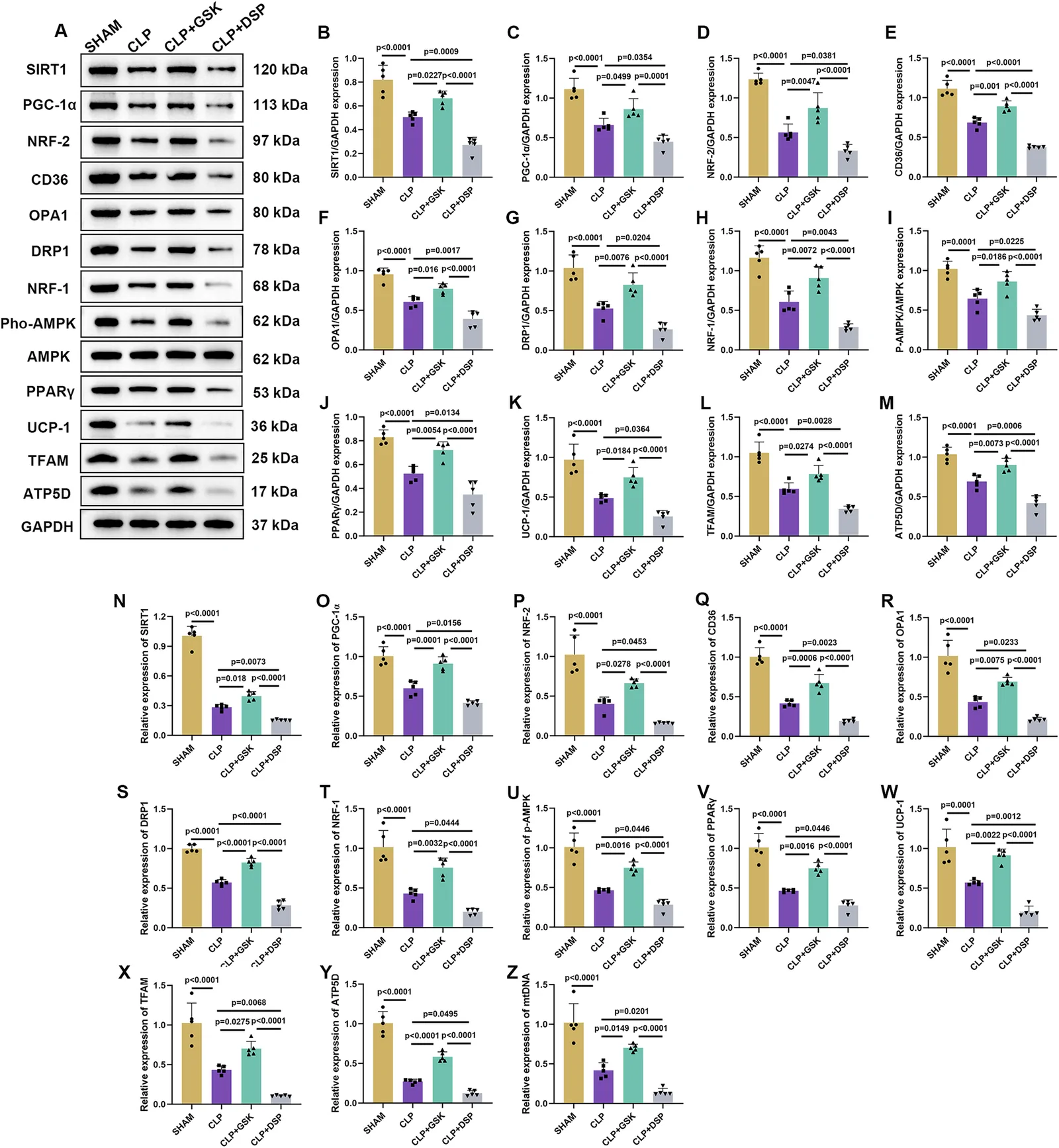
Modulation of the AMPK/SIRT1 signaling pathway to activate downstream targets affects SAW in CLP mice. **A**–**M** Western blot detection of protein expression levels of related signaling pathways in mouse muscle tissue and data analysis. **N**–**Z** RT-qPCR detection of transcript levels of genes in mouse muscle tissue. AMPK adenosine 5’-monophosphate (AMP)-activated protein kinase, SIRT1 silent information regulator 1, NRF-1/2 nuclearrespiratoryfactor1/2, OPA1 optic atrophy 1, DRP1 dynamin-related protein 1, TFAM mitochondrial transcription factor A, GAPDH glyceraldehyde-3-phosphate dehydrogenase, mtDNA mitochondrial DNA. Mean ± SD. *n* = 5.

Afterward, we used molecular experiments to verify the changes in protein levels. The ELISA results were consistent with the histological validation. The levels of ACH, PGC-1α, PPARγ, and CD36 were decreased in CLP mice compared to SHAM mice (Fig. 3A, E–G) (*P* < 0.05), whereas the levels of AchE, TNF-α, and IL-6 were gradually increased (Fig. 3B–D) (*P* < 0.05), and the content changes were more significant in CLP + DSP mice, and GSK621 intervention reversed the factor content changes. The results of immunohistochemistry (Fig. 3H–O) were consistent with the results of western blot/RT-qPCR (Fig. 4): compared with SHAM mice, the expression levels of proteins related to the energy metabolism pathway (AMPK/SIRT1/PGC-1α/PPARγ/NRF-1/NRF-2) and mitochondrial function (CD36/OPA1/DRP1/UCP-1/TFAM/ATP5D/mtDNA) were significantly lower in the muscle tissues of CLP mice (*P* < 0.05). Among them, CLP + DSP mice had lower protein expression levels than CLP mice, and GSK621 intervention reversed the protein expression levels. Thus, activation of the AMPK/SIRT1 signaling pathway can improve SAW in CLP mice.

### Inactivation of the PGC-1α/PPARγ pathway exacerbates muscle tissue lesions and mitochondrial dysfunction in CLP mice

**Fig. 5:**
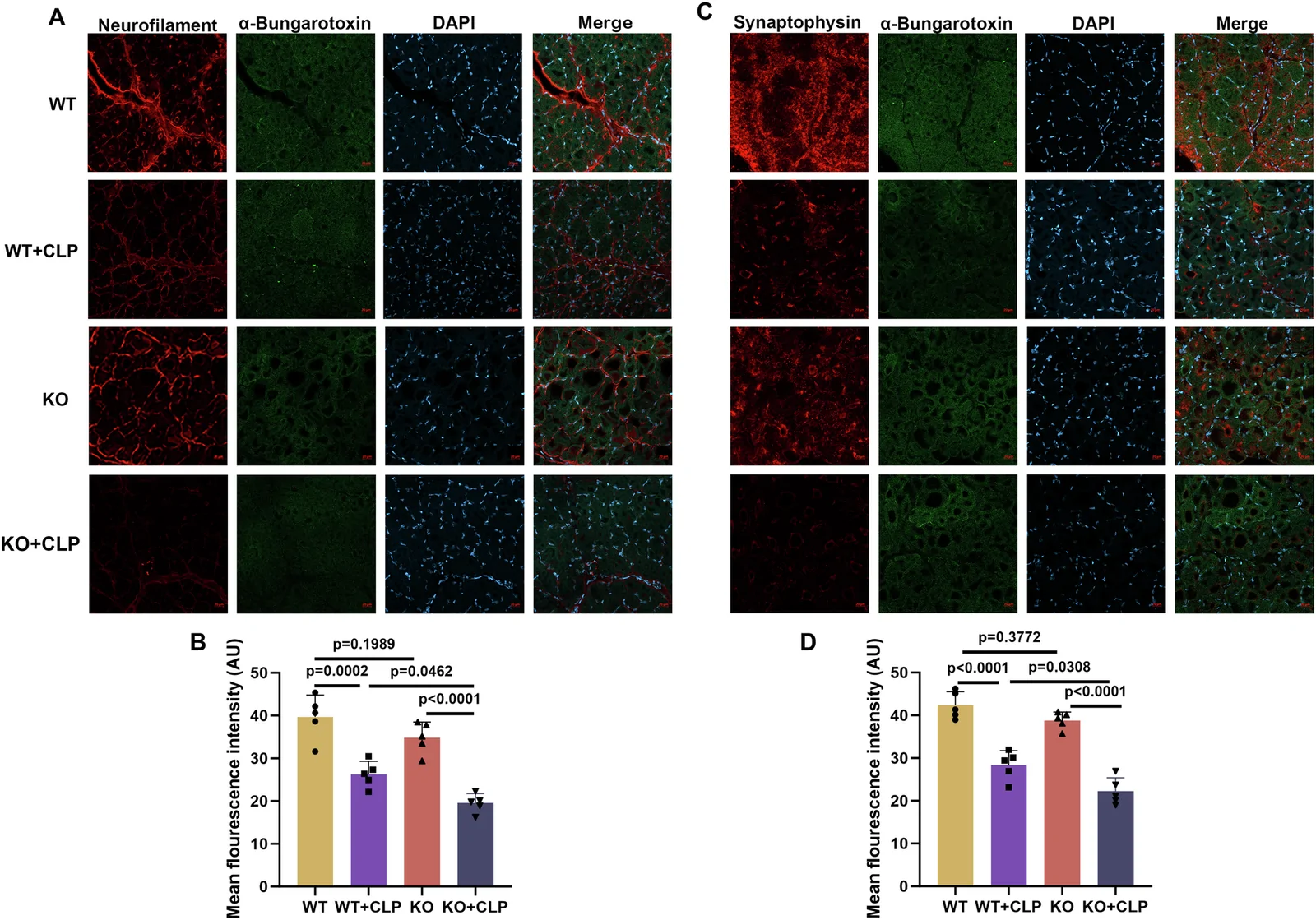
Deficiency of PGC-1α exacerbates neuromuscular dysfunction in CLP mice. **A**, **B** Neurofilament/α-Bungarotoxin immunofluorescence plots and data analysis. **C**, **D** Synaptophysin/α-Bungarotoxin immunofluorescence plots and data analysis. KO, Mice with knockout PGC-1α; WT, Knockout PGC-1 mice littermate wild mice; CLP, Mouse model of sepsis. Mean ± SD. *n* = 5. (scale bar, 20 μm).

To investigate the function of PGC-1α in muscle tissue during sepsis, we constructed a CLP model using transgenic mice with PGC-1α knocked out. The findings of immunofluorescence double labeling demonstrated a considerable decrease in NF/SYP expression in WT + CLP mice compared to WT mice (*P* < 0.05). Furthermore, the level of NF/SYP expression in muscle tissues of KO + CLP mice was significantly down-regulated compared to WT + CLP mice (Fig. 5A–D) (*P* < 0.05). The inhibition of neurofilament and synaptic protein activation is clearly observed when PGC-1α is knocked out.

**Fig. 6:**
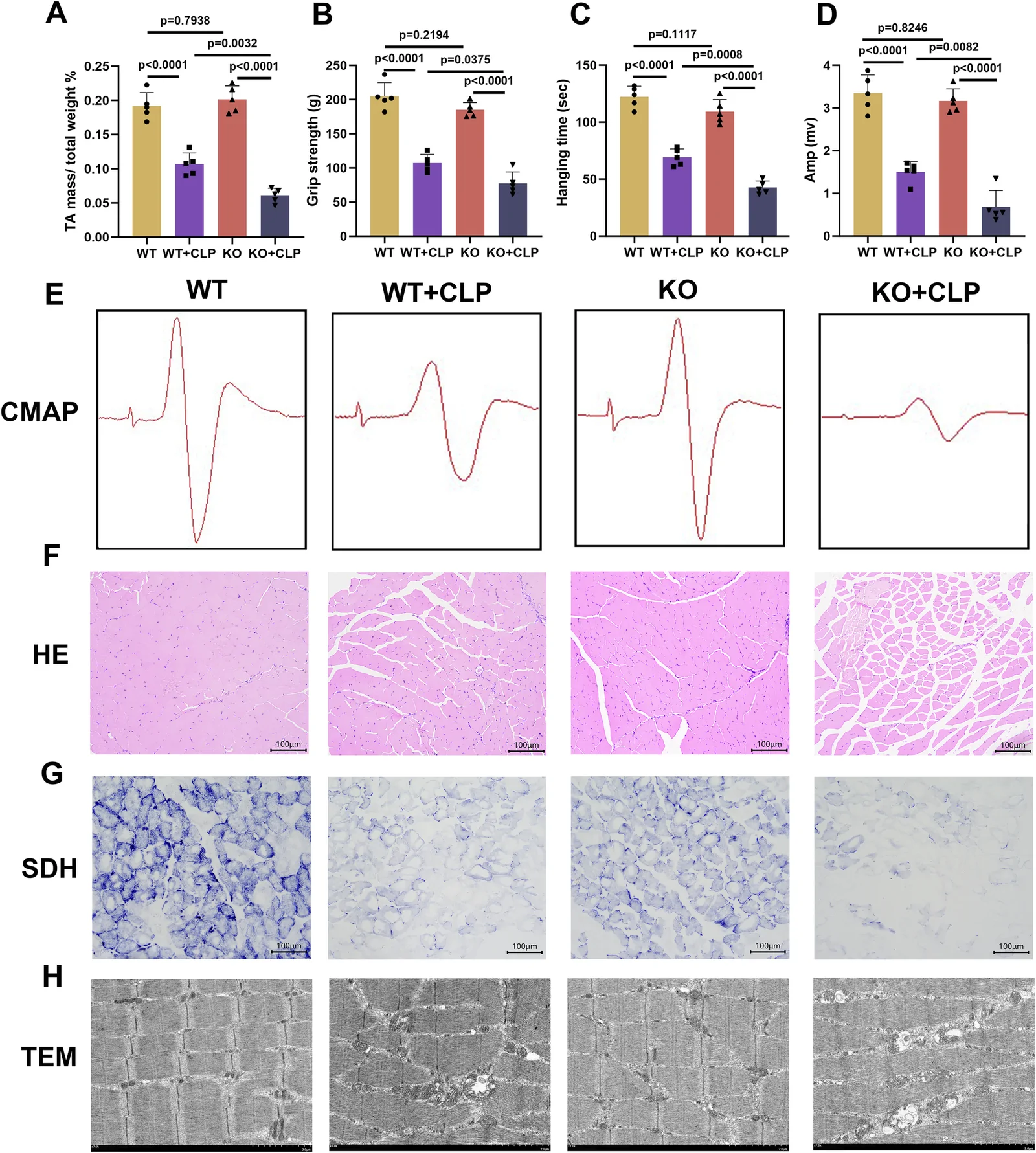
Deficiency of PGC-1α exacerbates SAW in CLP mice. **A** The TA mass/total weight of mice. **B** The grip strength of mice. **C** The hanging time of mice. **D**, **E** CMAP for the detection of muscle weakness status in mice and its quantitative analysis. **F** HE staining of mouse muscle tissue. **G** SDH staining of mouse muscle tissue. **H** Mitochondrial ultrastructure observed by TEM of mouse muscle tissue. TA tibialis anterior muscle, CMAP compound muscle action potential, HE hematoxylin-eosin, SDH succinate dehydrogenase, TEM transmission electron microscope; Mean ± SD. *n* = 5. (scale bar, HE/SDH, 100 μm; LFB, 50 μm; TEM, 2 μm).

**Fig. 7:**
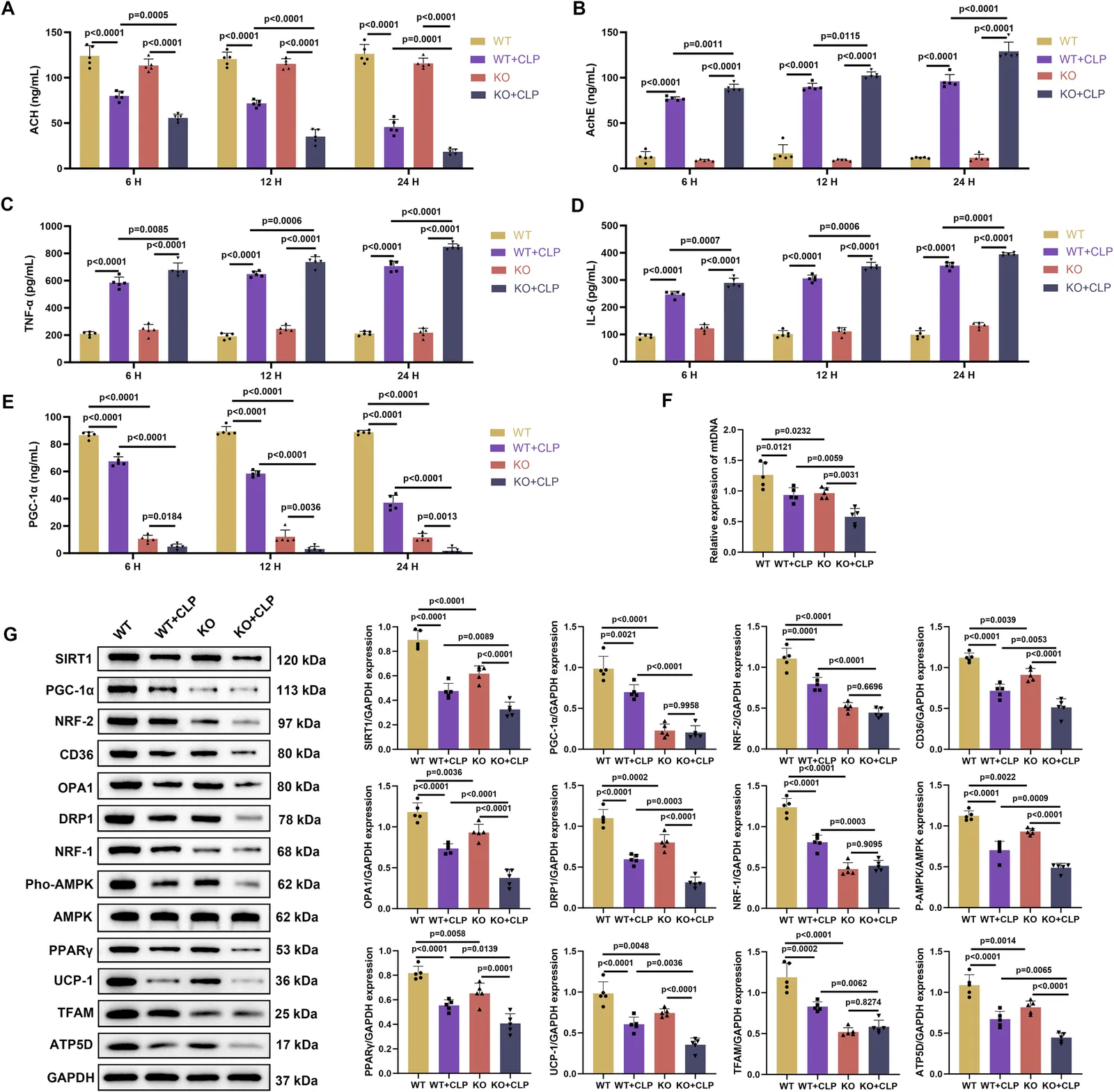
Deficiency of PGC-1α exacerbates SAW in CLP mice. **A**–**E** ELISA for determination of cytokine levels in mouse serum at different time points (6/12/24 h) after modeling. **F** RT-qPCR detection of mtDNA in mouse muscle tissue. **G** Western blot detection of protein expression levels of related signaling pathways in mouse muscle tissue and data analysis. Mean ± SD. *n* = 5.

Similarly, we detected the TA muscle content, grip strength, suspension time and CMAP detection of mice (Fig. 6A–E). The results showed that all the indicators of KO were lower than those of WT mice, indicating that PGC-1α associated with improvements. Following that, we confirmed that PGC-1α significantly improved SAW by muscle histological analysis. HE staining showed that WT mice showed regular arrangement of myofibres, normal myocyte structure, and clear transverse striations. KO mice had swollen myocytes with disorganized nuclei. The myocyte gaps were widened in WT + CLP mice. Multiple regions of myoplasmic staining became lighter and some of the transverse striations disappeared. The pathological changes were most severe in KO + CLP mice (Fig. 6F). The SDH staining results showed that the SDH content was significantly lower in KO mice compared with WT mice, and lower in WT + CLP mice compared with untreated mice (WT/KO), and almost absent in KO + CLP mice (Fig. 6G). Meanwhile, our TEM results showed that WT mice had uniform chromatin distribution in the nucleus, abundant perinuclear mitochondria, well-defined mitochondrial structure, neatly arranged myofilaments with abundant morphologically and structurally intact mitochondria in between, and intact myofilaments. Mitochondrial cristae of WT + CLP mice were broken, dissolved, and even formed blank areas. Mitochondrial damage was aggravated in KO + CLP mice (Fig. 6H). The ELISA results were in agreement with the histological verification was consistent with the fact that the content of ACH and PGC-1α decreased, while the content of AchE, TNF-α, and IL-6 factors increased in WT + CLP mice compared with that of untreated mice, and the change of the content was more significant in KO + CLP mice (Fig. 7A–E).

We used western blot and RT-PCR techniques respectively to detect and verify the expression levels of signaling pathway-related proteins and the expression status of mtDNA in mouse muscle tissues (Fig. 7F, G). Our results demonstrated that the expression levels of proteins related to the energy metabolism pathway (AMPK/SIRT1/PGC-1α/PPARγ/NRF-1/NRF-2) and mitochondrial function (CD36/OPA1/DRP1/UCP-1/TFAM/ATP5D) were significantly decreased in comparison to unmodelled mice (*P* < 0.05). Of these, the protein expression levels were reduced in KO + CLP mice compared to WT + CLP mice. The lack of PGC-1α clearly hinders the activation of NF and SYP, exacerbating muscle tissue damage and impairing mitochondrial function in CLP mice. This could be attributed to the deactivation of the PGC-1α/PPARγ pathway.

### Activation of AMPK/SIRT1 signaling pathway acts on PGC-1α/PPARγ to alleviate SAW in CLP mice

**Fig. 8:**
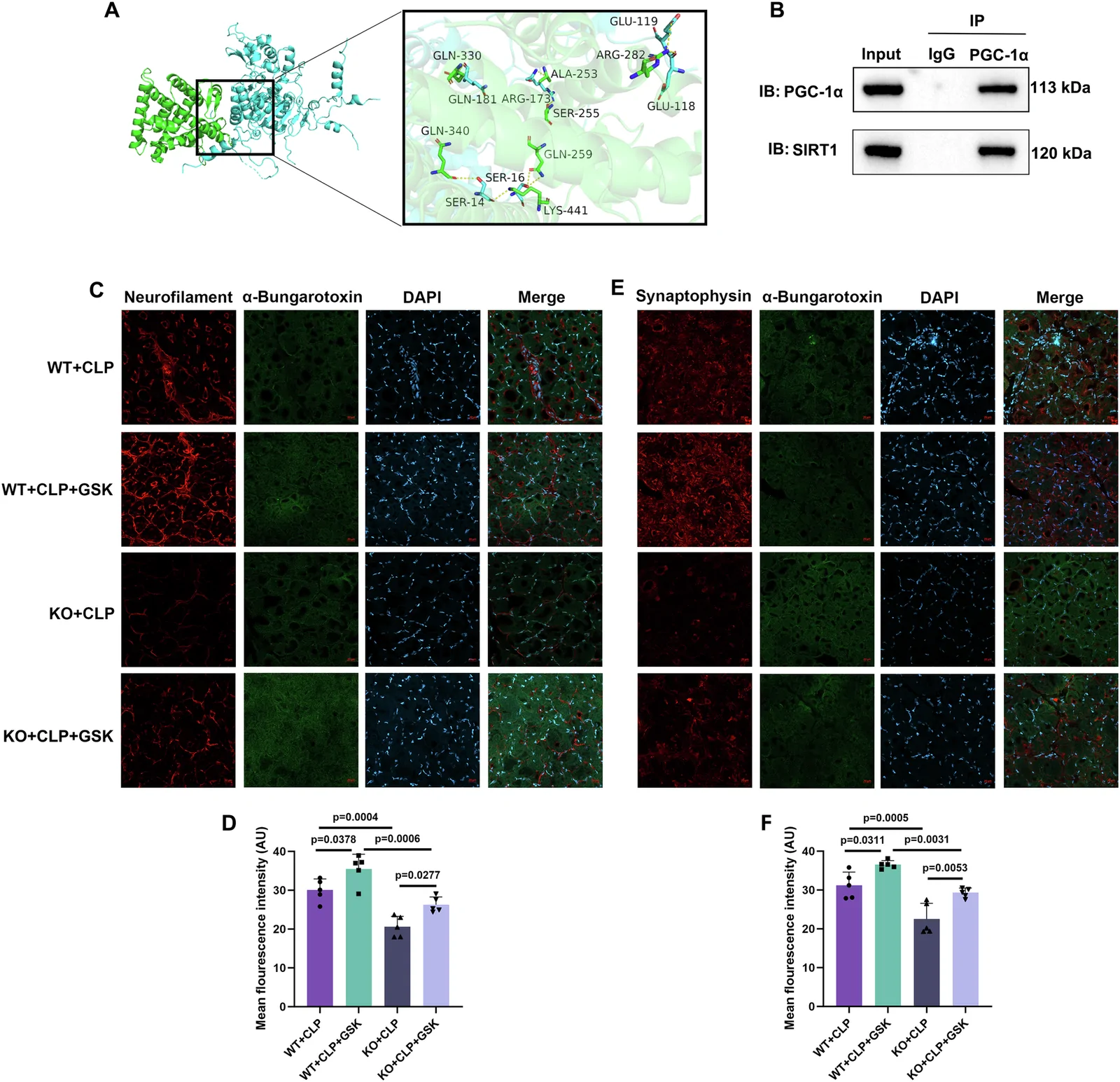
Activation of the AMPK/SIRT1 pathway alleviates neuromuscular dysfunction in KO mice. **A** The HDOCK online program was used to perform molecular docking experiments for predicting the binding ability between SIRT1 and PGC-1α. **B** Co-IP assay was performed to verify whether there is an interaction between SIRT1 protein and PGC-1α protein. **C**, **D** Neurofilament/α-Bungarotoxin immunofluorescence plots and data analysis. **E**, **F** Synaptophysin/α-Bungarotoxin immunofluorescence plots and data analysis. Mean ± SD. *n* = 5. (scale bar, 20 μm).

Our previous research demonstrated that activating the AMPK/SIRT1 signaling pathway improved muscle histopathology and mitochondrial dysfunction in CLP mice with SAW. Conversely, deactivating the PGC-1α/PPARγ pathway worsened these conditions. Therefore, we aimed to investigate if we could alleviate SAW in CLP mice by activating the AMPK/SIRT1 signaling pathway that acts on PGC-1α/PPARγ. First, the potential interaction between SIRT1 and PGC-1α was predicted using molecular docking technology, with the results presented in Fig. 8A. Analysis revealed the presence of multiple potential binding sites between SIRT1 and PGC-1α, with a docking score of -232.02 and a confidence score of 0.8376—indicating a very high binding potential between the two proteins. To further validate this predicted result, Co-IP assay was subsequently performed for empirical verification. The results confirmed that SIRT1 and PGC-1α can indeed interact with each other (Fig. 8B).

**Fig. 9:**
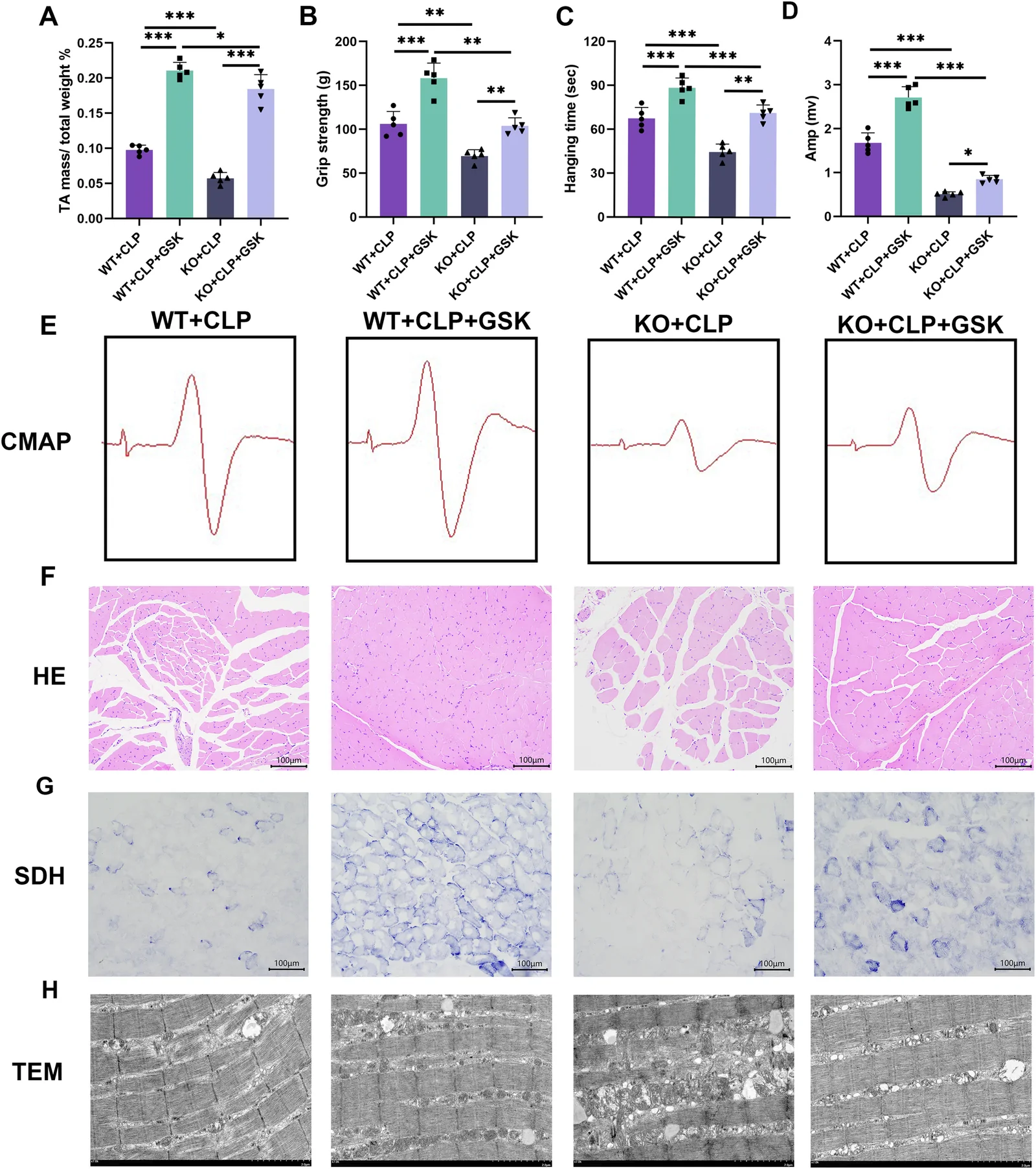
Activation of the AMPK/SIRT1 pathway alleviates SAW in KO mice. **A** The TA mass/total weight of mice. **B** The grip strength of mice. **C** The hanging time of mice. **D**, **E** CMAP for the detection of muscle weakness status in mice and its quantitative analysis. **F** HE staining of mouse muscle tissue. **G** SDH staining of mouse muscle tissue. **H** Mitochondrial ultrastructure observed by TEM of mouse muscle tissue. TA tibialis anterior muscle, CMAP compound muscle action potential, HE hematoxylin-eosin, SDH succinate dehydrogenase, TEM transmission electron microscope; Mean ± SD. *n* = 5. (scale bar, HE/SDH, 100 μm; LFB, 50 μm; TEM, 2 μm).

**Fig. 10:**
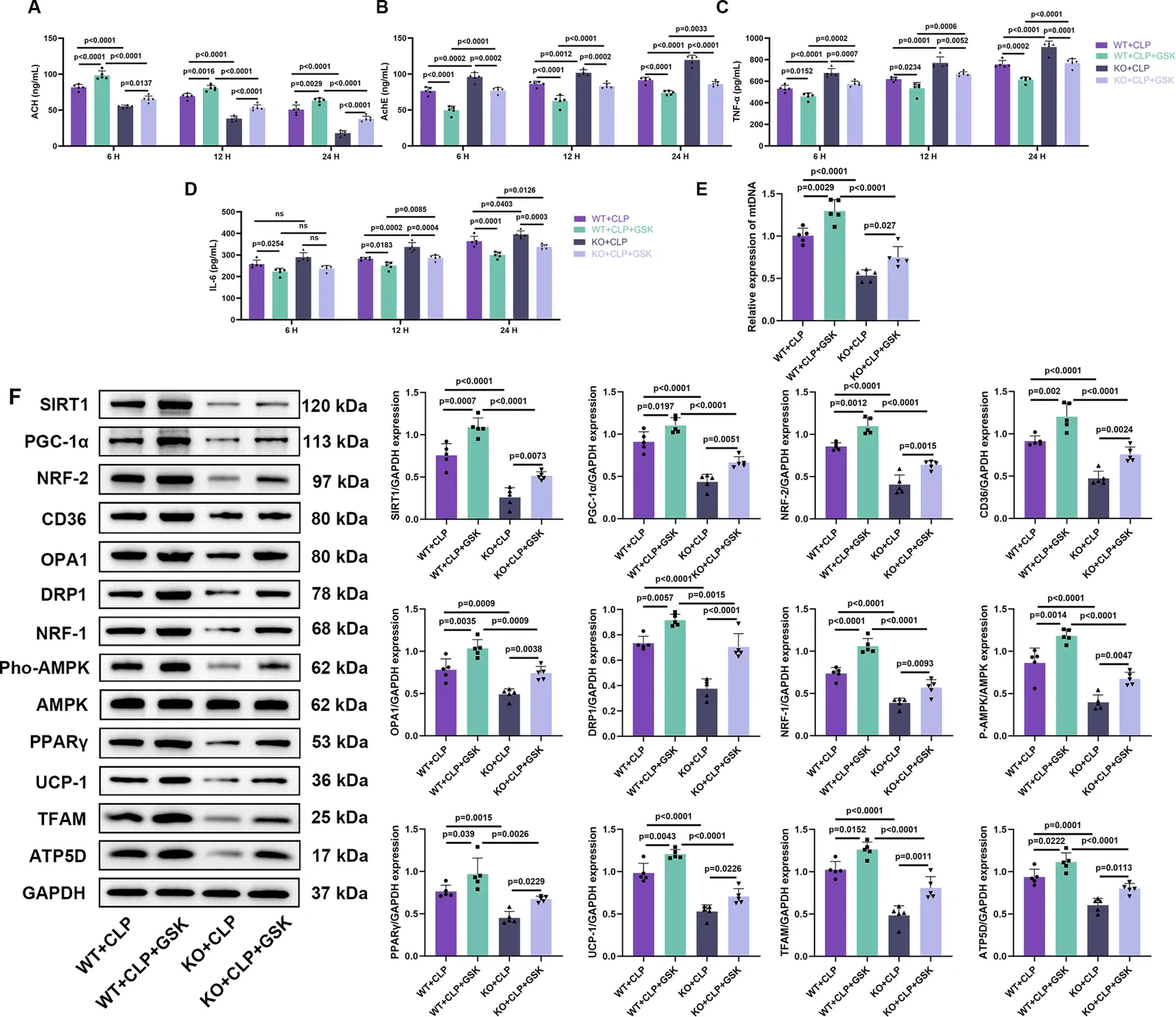
Activation of the AMPK/SIRT1 pathway alleviates SAW in KO mice. **A**–**D** ELISA for determination of cytokine levels in mouse serum at different time points (6/12/24 h) after modeling. **E** RT-qPCR detection of mtDNA in mouse muscle tissue. **F** Western blot detection of protein expression levels of related signaling pathways in mouse muscle tissue and data analysis. Mean ± SD. *n* = 5.

Immunofluorescence double labeling results demonstrated a sequential decrease in NF/SYP content in the muscles of WT + CLP mice and KO + CLP mice. However, GSK621 intervention effectively increased the NF/SYP content in the muscles (Fig. 8C–F). Behavioral and histological examinations revealed that the muscle pathology of WT + CLP and KO + CLP mice progressively increased, the SDH content decreased, and the mitochondrial shape and tissue continuously deteriorated. However, these effects were significantly reversed by the intervention of GSK621. (Fig. 9A–H). ELISA assays showed that as time extended, the blood levels of ACH in WT + CLP mice and KO + CLP mice declined dramatically (Fig. 10A) (*P* < 0.05). Additionally, the levels of AchE and inflammatory factors increased significantly (Fig. 10B–D) (*P* < 0.05). However, this effect was significantly reversed by GSK621 intervention. We conducted Western blot and RT-PCR analyses to assess the expression of pathway-associated factors. Consistent with previous findings, the levels of proteins associated with energy metabolism pathways (AMPK/SIRT1/PGC-1α/PPARγ/NRF-1/NRF-2) and mitochondrial function (CD36/OPA1/DRP1/UCP-1/TFAM/ATP5D/mtDNA) were progressively decreased (*P* < 0.05). However, treatment with GSK621 was able to restore the protein expression levels (Fig. 10E, F). This demonstrates that the activation of the AMPK/SIRT1 signaling pathway affects PGC-1α/PPARγ in order to relieve mitochondrial dysfunction in CLP mice.

## Discussions

Sepsis-Associated Weakness (SAW), a severe complication of sepsis, significantly compromises the recovery trajectory and long-term prognosis of afflicted patients, whereas its core molecular mechanisms have not been fully delineated [20–22]. In this study, we systematically dissected the regulatory network of the AMPK/SIRT1-PGC-1α/PPARγ signaling axis in the pathogenesis of SAW by combining pharmacological interventions with genetic knockout strategies. The core results revealed that activation of the AMPK/SIRT1 signaling pathway could target the PGC-1α/PPARγ cascade, thereby mitigating SAW symptoms in mice with CLP-induced sepsis. In contrast, PGC-1α knockout exacerbated skeletal muscle damage and mitochondrial dysfunction. Notably, AMPK activation still exerted a partial protective effect even under the condition of PGC-1α deficiency, and the amelioration of muscle function was temporally correlated with the resolution of systemic inflammation. Collectively, these findings not only deepen the mechanistic understanding of SAW pathogenesis, but also provide a novel theoretical basis for the development of therapeutic strategies targeting the AMPK/SIRT1-PGC-1α/PPARγ regulatory axis.

A key breakthrough of the present study is that PGC-1α knockout significantly downregulates the expression levels of p-AMPK and SIRT1 in CLP mice. This finding overturns the traditional unidirectional regulatory paradigm and reveals a bidirectional interactive regulatory mechanism between PGC-1α and the AMPK/SIRT1 pathway. As a core regulator of mitochondrial biogenesis and metabolic homeostasis, PGC-1α has long been recognized as a downstream effector of AMPK and SIRT1 [23–25]. Activation of AMPK promotes the transcriptional activation of PGC-1α by phosphorylating cAMP response element binding protein CREB, whereas SIRT1 enhances the functional activity of PGC-1α through deacetylation modification [15, 26, 27]. However, the results of the present study demonstrated that PGC-1α deficiency reversely inhibits AMPK phosphorylation and SIRT1 expression, suggesting a feedback regulatory loop between the two.

We speculate two potential underlying mechanisms: First, PGC-1α knockout induces severe mitochondrial dysfunction, leading to intracellular energy homeostasis imbalance and metabolic disturbance. Sustained abnormal energy metabolism may suppress the activation of upstream kinases of AMPK, and enhance the activity of AMPK phosphatases, ultimately resulting in a reduction in p-AMPK levels [28, 29]. Second, as a transcriptional coactivator, PGC-1α lacks a direct DNA binding domain [30], but can indirectly regulate the transcriptional expression of SIRT1 by forming functional complexes with forkhead box O family (FoxO) transcription factors. Previous studies have confirmed that PGC-1α binds to FoxO1 and co-localizes to the SIRT1 promoter region, thereby enhancing the transcriptional activity of SIRT1 by improving the assembly efficiency of the transcription complex [31]. PGC-1α deficiency disrupts the formation of this regulatory complex, leading to the downregulation of SIRT1 expression. In addition, loss of PGC-1α mediated mitochondrial quality control exacerbates the production of reactive oxygen species (ROS), which inactivates SIRT1 through oxidative modification of key amino acid residues [32–34]. Collectively, the present study clarifies that PGC-1α forms a bidirectional regulatory network with the AMPK/SIRT1 pathway and establishes the central role of PGC-1α as a molecular hub integrating energy metabolism and inflammatory regulation.

Although PGC-1α knockout resulted in the downregulation of p-AMPK and SIRT1 expression, the findings of the present study demonstrated that treatment with the AMPK agonist GSK621 still improved muscle function, restored mitochondrial structure, and preserved neuromuscular transmission in CLP mice with PGC-1α knockout, suggesting that AMPK exerts its protective effects via both PGC-1α-dependent and PGC-1α-independent mechanisms. On the one hand, even in PGC-1α knockout mice, trace amounts of PGC-1α isoforms (e.g., PGC-1α2) or homologous proteins (e.g., PGC-1β) may remain, which can be activated by AMPK to partially restore mitochondrial biogenesis and function [35, 36]. On the other hand, AMPK can directly regulate multiple downstream targets involved in the maintenance of skeletal muscle homeostasis in a PGC-1α-independent manner. For instance, AMPK can inhibit the mammalian target of rapamycin (mTOR) signaling pathway to attenuate skeletal muscle protein degradation; meanwhile, it can phosphorylate mitochondrial fission and fusion-related proteins (e.g., DRP1, OPA1) to optimize mitochondrial dynamic balance [37–40]. The TEM data in the present study revealed that GSK621 alleviated mitochondrial swelling and cristae disruption in PGC-1α knockout mice, providing direct evidence for this mechanism. Furthermore, AMPK activation can suppress the activity of the NF-κB pathway, reducing the synthesis and release of pro-inflammatory cytokines (TNF-α, IL-6), thereby mitigating inflammation-mediated myocyte injury and neuromuscular junction dysfunction. These PGC-1α-independent pathways act in functional complementation with the PGC-1α-dependent regulatory axis, which molecularly accounts for the core mechanism underlying the preserved protective efficacy of AMPK activation under PGC-1α-deficient conditions. This finding holds important clinical implications, indicating that AMPK agonists may still confer therapeutic benefits for SAW patients with reduced PGC-1α expression induced by genetic polymorphisms, severe sepsis-triggered metabolic reprogramming, or other pathological factors.

Through sequential ELISA detections at 6, 12, and 24 h post-CLP surgery, combined with histological and functional evaluations at the experimental endpoint, the present study systematically delineated the temporal association between systemic inflammation and skeletal muscle injury. Dynamic detection results revealed that plasma levels of pro-inflammatory cytokines (TNF-α, IL-6) in CLP mice exhibited a time-dependent elevation within 6–24 h, accompanied by a progressive reduction in ACh levels and sustained upregulation of AchE activity. This finding indicates that the processes of systemic inflammatory outburst and neuromuscular junction dysfunction are initiated as early as the acute phase of sepsis.

Collectively, these early alterations in inflammatory responses and neurobiochemical markers were highly consistent with the severe skeletal muscle atrophy, mitochondrial structural damage, neuromuscular junction disruption, and decreased muscle strength observed at the experimental endpoint, thereby uncovering a clear pathophysiological temporal cascade: early-onset and progressively aggravating systemic inflammation serves as the initiating factor for SAW, which subsequently drives the progressive aggravation of skeletal muscle morphological and functional impairments during disease progression. Intervention with the AMPK agonist GSK621 markedly abrogated the time-dependent elevation of pro-inflammatory cytokines within 6–24 hours and ultimately reversed the skeletal muscle dysfunction and structural damage detected at the endpoint. This definitive temporal correlation strongly confirms that the inhibition of early inflammatory progression by AMPK activation is a critical prerequisite for the subsequent repair of muscle pathological injuries and functional improvement, rather than a synchronous effect. Consistent with previous research findings [41, 42], the present study further validates that the uncontrolled inflammatory response in the early phase of sepsis is a core upstream driver for the development and progression of SAW. Targeted intervention of early inflammatory signaling pathways can effectively block the pathological cascade leading to persistent skeletal muscle injury.

By delineating the bidirectional regulatory crosstalk between PGC-1α and the AMPK/SIRT1 pathway in SAW, the present study has broadened the cognitive boundaries of previous research. Previous studies have predominantly focused on the positive regulatory activation of PGC-1α by the AMPK/SIRT1 pathway [43, 44]. In contrast, the present study demonstrates for the first time that PGC-1α is an indispensable molecule for maintaining the basal activity of the AMPK/SIRT1 pathway, which further refines the molecular regulatory network of SAW and establishes PGC-1α as a central hub integrating energy metabolism and inflammatory regulation.

Meanwhile, the preserved protective effect of AMPK activation in PGC-1α-knockout mice suggests that the clinical application scope of AMPK agonists may be wider than previously recognized, and such agonists may still exert therapeutic value even in SAW patients with impaired PGC-1α expression. In clinical practice, SAW remains a major clinical challenge lacking effective interventions [5]. The findings of the present study indicate that AMPK agonists (e.g., GSK621) may serve as potential therapeutic candidates for SAW by simultaneously targeting three core pathological processes: inflammatory response, mitochondrial dysfunction, and skeletal muscle protein metabolic imbalance. Furthermore, the temporal correlation between early inflammatory inhibition and subsequent improvement in muscle function highlights the importance of implementing early intervention in sepsis patients to prevent irreversible muscle injury and functional impairment. Targeted clinical trials are warranted in the future to systematically evaluate the efficacy of AMPK agonists in improving muscle function and long-term prognosis in SAW patients, and to optimize the intervention timing, dosage, and treatment course.

Although the present study provides novel insights into the role of the AMPK/SIRT1-PGC-1α/PPARγ axis in SAW, several limitations should be noted. First, the CLP mouse model fails to fully recapitulate the pathophysiological complexity of human sepsis, and further validation in clinically relevant models is required to enhance translational value. Second, the gene knockdown strategy and AMPK modulators used in this study are experimental tools with obvious clinical constraints, such as delivery challenges, off-target effects, and unknown long-term safety, which restrict direct clinical translation. Third, only SIRT1 expression was detected without evaluating its enzymatic activity that determines its biological function. Fourth, the morphological changes of neuromuscular junctions, a core pathological feature of SAW, were not assessed. Fifth, the precise molecular mechanism of the bidirectional feedback loop between PGC-1α and AMPK/SIRT1 remains to be fully clarified. Finally, the dynamic analysis was limited to three early time points for inflammatory detection and a single muscle functional endpoint, and a more comprehensive sampling strategy would help better characterize the temporal coupling among inflammatory resolution, mitochondrial repair, and skeletal muscle function recovery.

## Conclusion

The present study demonstrates that the AMPK/SIRT1-PGC-1α/PPARγ axis functions as a central regulatory signaling cascade in the pathogenesis of SAW. PGC-1α ablation impairs the activation of the AMPK/SIRT1 pathway via a negative feedback loop, whereas pharmacological activation of AMPK exerts protective effects on skeletal muscle through both PGC-1α dependent and PGC-1α independent mechanisms. A clear temporal correlation was identified between the amelioration of muscle function and the early suppression of systemic inflammation, where effective blockade of the progressive inflammatory elevation within 6–24 h after sepsis onset precedes and determines the ultimate recovery of skeletal muscle structure and function. Collectively, these findings provide novel mechanistic insights into the molecular pathogenesis of SAW and establish a vital theoretical framework for the development of AMPK targeted therapeutic strategies for the clinical management of sepsis-related complications.

## Materials and methods

### Animals

We commissioned Cyagen Biotechnology Ltd (SCXK(SU)2020-0008, CHN) to construct and breed PGC-1α knockout mice (KO) and their wild-type mice (WT, C57BL/6 J). All mice were housed in the SPF animal laboratory with 50–60% humidity, 22–25 °C, a 12-h light/dark cycle, and ad libitum food and water intake. All animal procedures were performed in strict accordance with then ARRIVE guidelines, the National Institutes of Health guide for the care and use of Laboratory animals (NIH Publications No. 8023, revised 1978), and the recommendations of the Guidelines for Care and Use of Laboratory Animals, and the experiments were approved by Medical Ethics Committee of Weihai Municipal Hospital (Protocol Number: 2022095).

### Construction and grouping of sepsis mouse model

Mice weighing 21–25 g were selected, and the animals were acclimatized for 3–5 days and fasted for 12 h before the experiment. Septic mice were constructed by cecum ligation puncture (CLP) method [45]. The animals were anesthetized by intraperitoneal injection of sodium pentobarbital (P3761, Sigma, USA) at a dose of 0.05 mg/g of mouse body weight. The animals were fixed supine on a surgical plate. The abdomen was disinfected and depilated, and a longitudinal incision of 2 cm was made along the midline of the abdomen of the mice. A 2 cm long longitudinal incision was made through the median abdominal incision to separate the cecum and the cecum was ligated with 4–0 silk suture at one-third of its distal end, followed by a single puncture on the antimesenteric wall of the ligated cecal segment using an 18 G needle. A small amount of intestinal contents was gently extruded from the puncture site, after which the cecum was carefully returned to its original anatomical position in the abdominal cavity, and the abdominal incision was closed layer by layer.

### Animal grouping and interventions

This study was conducted in three parts to systematically elucidate the regulatory role of the AMPK/SIRT1-PGC-1α/PPARγ axis in SAW. A total of 10 mice per group survived until the experimental endpoint, and the survival rate of the CLP-induced sepsis model was approximately 60% (Supplementary Fig. 1). In the first part, to explore the role of the AMPK/SIRT1 signaling pathway in SAW, mice were randomly divided into four groups: sham-operated group (Sham), CLP-induced sepsis model group (CLP), CLP group treated with the AMPK agonist GSK621 (CLP + GSK), and CLP group treated with the AMPK inhibitor Dorsomorphin (CLP + DSP). In the second part, to investigate the function of PGC-1α in SAW, wild-type (WT) and PGC-1α knockout (KO) mice were assigned into four groups: WT group, KO group, WT + CLP group, and KO + CLP group. In the third part, to further verify whether AMPK/SIRT1 mediates the development of SAW by regulating the PGC-1α/PPARγ axis, four groups were established: WT + CLP + GSK group, WT + CLP group, KO + CLP + GSK group, and KO + CLP group.

The CLP procedure was performed as previously described to establish the mouse sepsis model. Mice in the Sham group underwent the same surgical procedures as those in the CLP group, except that cecal puncture was not performed. GSK621 (GSK, HY-100548, MCE, USA, 30 mg/kg) [46] and DSP (HY-13418A, MCE, USA, 50 mg/kg) [47] were administered via intramuscular injection into the bilateral tibialis anterior muscles at 1 h after CLP, and the injection was repeated every 24 h until the mice were sacrificed. All intramuscular injections were targeted at the bilateral tibialis anterior muscles to ensure adequate drug distribution in the target skeletal muscle tissue and improve experimental reproducibility. Mice in the control groups, including the Sham, WT, and KO groups, were given an equal volume of vehicle solution using the same injection protocol. At the end of the experiment, plasma and tibialis anterior (TA) muscles of the mice were collected for subsequent analyses. Animals were randomly assigned to each group via a random number table. A double-blinding strategy was adopted in the study: the operators for modeling/administration and the investigators for data detection/analysis were both blinded to group allocation until data analysis was completed, to avoid experimental bias.

### Compound muscle action potential (CMAP)

The mice were anesthetized, and after skin preparation, freeing the sciatic nerve, and isolating the gastrocnemius muscle, the stimulator and recording device were attached. The effect of stimulation intensity on muscle contraction was observed (RM6240EC Multi-Channel Physiological Signal Acquisition and Processing System, Chengdu Instrument Factory, CHN). Software settings: Signal selection - check the first box to set myoelectricity - check the second box to set tension - two-channel traps were turned on - stimulus triggering was changed to continuous sampling.

### Muscle function

The grip strength experiment of mice was conducted using a grip dynamometer (Shanghai Biotechnology Co., LTD., China). The specific operation is to let the mouse’s claws grasp the grid, and then slowly drag it backward until it can no longer grasp it. The maximum tension values in the five tests were recorded in grams as one measurement. During the hanging experiment, the mice were placed on an elevated inverted grid 80 cm above the ground, and the suspension time was measured. Make ten measurements and take the maximum value.

### ELISA assay

Plasma and tibialis anterior muscle tissues were harvested from mice at 6, 12, and 24 hours for subsequent ELISA analysis, and plasma samples were specifically used for the detection of inflammatory factors. Using ELISA kits: PGC-1α (E1160m, ELAab, CHN); Tumor Necrosis Factor alpha (TNF-α, Tumor Necrosis Factor alpha, PT512, Beyotime, CHN); Interleukin 6 (IL-6, PI326, Beyotime, CHN); Cluster of differentiation 36 (CD36, ab100673, Abcam, USA); Acetylcholine (ACH, ml401805, mlbio CHN); Acetylcholinesterase (AchE, ml037240, mlbio, CHN), and PPARγ (E-EL-M0893, Elabscience, CHN) were subjected to enzyme-linked immunosorbent assay for the detection of levels.

### Hematoxylin-eosin (HE) staining

After paraffin sections of mouse TA muscle were dewaxed and hydrated with xylene and ethanol in a concentration gradient, the sections were incubated with HE staining solution (G1120, Solarbio, CHN) for 5 min at room temperature, and then dehydrated and transparent using ethanol and xylene. Finally, after sealing the sections with neutral gel, images of the corresponding pathological changes were obtained using a microscope (CX31, OLYMPUS, JP).

### Transmission electron microscopy (TEM)

Mouse tissue sections were fixed in 2.5% glutaraldehyde and stored at 4°C, fixed in 1% osmium acid for 2 h and then dehydrated with ethanol and acetone in a concentration gradient, embedded in Epon812 epoxy resin (E8000, HEAD, CHN), polymerized for 8 h in a warm box at 60°C, and observed by transmission electron microscopy (JEM-1400Flash, JEOL, JP) after double staining of ultrathin sections.

### Immunofluorescence & succinic dehydrogenase (SDH) staining

TA muscle tissue was taken, embedded using freezing embedding agent, sectioned, and rinsed. Sections were incubated with goat serum (005-000-121, Jackson, USA) for 1 h at room temperature, after which primary antibodies were added dropwise including neurofilament (1:500, ab7794, Abcam, UK), synaptophysin (1:500, ab32127, Abcam, UK), and α-bungarotoxin (1:200, HY-P1264, MCE, USA), and the sections was incubated overnight at 4°C. Subsequently, the tissues were incubated with the corresponding secondary antibody (1:200, AS001/AS011, ABclonal, CHN) at room temperature for 1 h. The tissues were stained with DAPI solution (C0065, Solarbio, CHN) for 1 h. Dropwise addition of Anti-fluorescence Burst Sealer (P0131, Beyotime, CHN) sealed the films, which were then stained in fluorescence microscope (DM1000, Leica, GER) and photographed. Fluorescence intensity analysis was performed using Image J software. Frozen sections were immersed in SDH action solution (R23066, SAINT-BIO, CHN) for 15 min and then rinsed, fixed, hydrated with ethanol, and then the sections were sealed with neutral adhesive, and images of the corresponding pathological changes were obtained using a microscope (DM2000, Leica, GER).

### Luxol Fast Blue (LFB) staining

The paraffin sections of sciatic nerve were dewaxed and hydrated with xylene and ethanol in a concentration gradient, then Luxol Fast Blue staining solution (G3245, Solarbio, CHN) was added for room temperature staining, and then Luxol differentiation solution was added to differentiate the colors, and ethanol and xylene were added for dehydration and transparency of the sections. Finally, images of the corresponding pathological changes were obtained using a microscope (CX31, OLYMPUS, JP).

### Western blot

Total proteins were extracted from the tibialis muscle of each group of mice separately, and the protein concentration was determined by BCA kit (A53225, Thermo Fisher, USA). The proteins were separated by SDS-PAGE, transferred to a PVDF membrane (88585, Thermo Fisher, USA), and incubated with rapid blocking solution (P0252, Beyotime, CHN) for 15 min and incubated overnight at 4 °C with different primary antibodies: AMPK (1:1000, #2532S, CST, USA); Pho-AMPK (1:1000, #2535S, CST, USA); CD36 (1:1000, #28109S, CST, USA); Dynamin-related protein 1 (DRP1, 1:1000, #8570S, the CST, USA); Glyceraldehyde-3-phosphate dehydrogenase (GAPDH, 1:1000, #2118S, CST, USA); Nuclear respiratory factor 1 (NRF-1, 1:1000, #46743S, CST, USA); Nuclear respiratory factor 2 (NRF-2, 1:1000, #12721S, CST, USA); Optic Atrophy 1 (OPA1, 1:1000, #80471S, CST, USA); PPARγ (1:1000, #2435S, CST, USA); SIRT1 (1:1000, #9475S, CST, USA); Uncoupling Protein 1 (UCP-1, 1:1000, #72298S, CST, USA); PGC-1α (1:1000, ab313559, Abcam, USA); ATP Synthase 5 Delta (ATP5D, 1:5000, ab174438, Abcam, UK); Mitochondrial transcription factor A (TFAM, 1:5000, 22586-1-AP, Proteintech, CHN). Subsequently, the protein membranes were incubated with the corresponding secondary antibodies (1:2000, AS014/AS003, ABclonal, CHN) at room temperature for 1 h. The protein membranes were processed by chemiluminescence detection kit (Advansta, Menlo Park, CA), and the blots were displayed in chemiluminescence imager (JY-Clear ECL, JUNYI, CHN), and analysed by the ImageJ software to analyze gray values for quantitative analysis of protein expression.

### Immunohistochemistry

Tissue sections were dewaxed and hydrated followed by high temperature antigen repair, followed by endogenous occlusion using 3% H_2_O_2_ (88597, Merck, USA) and exogenous occlusion using 5% BSA (V900933, Sigma, USA), and at the end of the occlusion dropwise addition of diluted antibodies (ATP5D, 1:250, ab97491, Abcam, UK; UCP-1, 1:500, ab10983, Abcam, UK; PGC-1α, 1:500, ab191838, Abcam, UK; PPARγ, 1:200, ab310323, Abcam, UK) on the sections and stored at 4 °C overnight, primary antibody incubation was completed by secondary antibodies (1:200, #8114S CST, USA) for 1 h. Subsequently, the sections were stained, dehydrated, and transparently sealed, and the protein expression was observed under a microscope (OLYMPUS, JP, CX31), and the data were analyzed by Image J software.

### RT-qPCR

Total RNA was isolated using TRIzol reagent (15596026CN, Invitrogen, USA) according to the manufacturer’s recommendations. First Strand cDNA synthesis was performed using Synthesis Kit (11917020, Invitrogen, USA) as described previously. For Real-time PCR, the expressions of the genes were assessed by PCR amplification with Bio-Rad CFX96 real-time detection system (USA) using SYBR Green Master Mix kit (A46110, Invitrogen, USA). The primer sequences were shown in Table 1.

**Table 1:** Primer sequence.

Gene name	Primer sequence
AMPK	forward primer: 5′- TTGAAACCTGAAAATGTCCTGCT-3′ reverse primer: 5′- GGTGAGCCACAACTTGTTCTT-3′
CD36	forward primer: 5′- ATGGGCTGTGATCGGAACTG-3′ reverse primer: 5′- TTTGCCACGTCATCTGGGTTT-3′
DRP1	forward primer: 5′- CGGAAACGGCCTCATGTGT-3′ reverse primer: 5′- CGCGTCGTGTACTCATCCAA-3′
NRF-1	forward primer: 5′- AGCACGGA GTGACCCAAAC-3′ reverse primer: 5′- AGGATGTCCGAGTCATCATAAGA-3′
NRF-2	forward primer: 5′- TCAGCGACGGAAAGAGTATGA-3′ reverse primer: 5′- CCACTGGTTTCTGACTGGATGT-3′
OPA1	forward primer: 5′-TGTGAGGTCTGCCAGTCTTTA-3′ reverse primer: 5′-TGTCCTTAATTGGGGTCGTTG-3′
PPARγ	forward primer: 5′-GGGATCAGCTCCGTGGATCT-3′ reverse primer: 5′-TGCACTTTGGTACTCTTGAAGTT-3′
SIRT1	forward primer: 5′-TAGCCTTGTCAGATAAGGAAGGA-3′ reverse primer: 5′-ACAGCTTCACAGTCAACTTTGT-3′
UCP1	forward primer: 5′-GTGAACCCGACAACTTCCGAA-3′ reverse primer: 5′-TGCCAGGCAAGCTGAAACTC-3′
PGC-1α	forward primer: 5′-TCTGAGTCTGTATGGAGTGACAT-3′ reverse primer: 5′-CCAAGTCGTTCACATCTAGTTCA-3′
ATP5D	forward primer: 5′-GGAG CGAGATCCCTCCAAAAT-3′ reverse primer: 5′-GGCTGTTGTCATACTTCTCATGG-3′
TFAM	forward primer: 5′-ATGGCGTTTCTCCGAAGCAT-3′ reverse primer: 5′-TCCGCCCTATAAGCATCTTGA-3′
mtDNA (Mitochondrial DNA)	forward primer: 5′-TTCTCCGACGTGCATATCCC-3′ reverse primer: 5′-GCGCTTCTTTTCTGTACCTTCT-3′
GAPDH	forward primer: 5′-ATGACTCTACCCACGGCAAG-3′ reverse primer: 5′-GGAAGATGGTGATGGGTTTC-3′

### Molecular docking

In this study, the online tool HDOCK (available at http://hdock.phys.hust.edu.cn/) was employed to perform molecular docking between PGC-1α and SIRT1 structures. Specifically, a fast Fourier transform (FFT) algorithm was utilized to search for potential binding models of the two proteins. Subsequently, a knowledge-based scoring function was applied to evaluate the sampled binding conformations, enabling the identification of favorable interaction modes. Finally, PyMOL software was used for visualization and plotting analysis to characterize the detailed intermolecular interactions between PGC-1α and SIRT1.

### Co-immunoprecipitation (Co-IP)

A total of 500 μg of total protein extract was mixed with 1 μg anti-PGC-1α specific antibody (66369-1-Ig, Proteintech, CHN); an equal-volume negative control was set up by adding the same amount of IgG (B900620, Proteintech, CHN) instead. Both groups were incubated on a slow shaker at 4 °C overnight. The next day, 30 μL pre-blocked (with 1% BSA in TBST) Protein A/G agarose beads (10004D, Thermo Fisher, USA) were added to each system, followed by further incubation at 4 °C for 2–4 h. Agarose bead precipitates were collected via centrifugation (3000 × *g*, 5 min, 4 °C) and gently washed 4–5 times with pre-cooled TBST. 2× SDS loading buffer was added to the precipitates, which were then boiled for 10 minutes. After re-centrifugation, the supernatant was collected for Western Blot detection.

### Statistical analysis

All data are expressed as mean ± standard (SD). One-way analysis of variance (ANOVA) was used for statistical analysis of multiple comparisons, followed by Tukey’s post-hoc test for multiple comparison analysis. *P* < 0.05 was considered statistically significant. Statistical analysis was carried out using the statistical software GraphPad Prism 8.0 (GraphPad Software, San Diego, CA, USA).

## Supplementary information

Full uncropped Gels and Blots images

Supplementary Figure

## Data Availability

The datasets used or analyzed during the current study are available from the corresponding author on reasonable request.
