# Supplementary material for: AMPK/ SIRT1 signaling pathway activation acts on PGC-1α/ PPARγ to alleviate sepsis-acquired weakness
Source: Cell Death Discov. 2026 Jun 23;12:354. doi: 10.1038/s41420-026-03212-w (PMC13542166; doi:10.1038/s41420-026-03212-w)
Supplement: Supplementary file 1 [file 41420_2026_3212_MOESM1_ESM.docx]

Fig 4

STRIT 1 PGC-1α

NRF-2 CD36

OPA1 DRP1

NRF-1 Pho-AMPK

AMPK PPARγ

UCP-1 TFAM

ATP5D GAPDH

Fig 7

SIRT1 PGC-1α

NRF-2 CD36

OPA1 DRP1

NRF-1 Pho-AMPK

AMPK PPARγ

UCP-1 TFAM

ATP5D GAPDH

Fig 8

 SIRT1 PGC-1α

Fig 10

SIRT1 PGC-1α

NRF-2 CD36

OPA1 DRP1

NRF-1 Pho-AMPK

AMPK PPARγ

UCP1 TFAM

ATP5D GAPDH
