# Supplementary material for: AMPK/ SIRT1 signaling pathway activation acts on PGC-1α/ PPARγ to alleviate sepsis-acquired weakness
Source: Cell Death Discov. 2026 Jun 23;12:354. doi: 10.1038/s41420-026-03212-w (PMC13542166; doi:10.1038/s41420-026-03212-w)
Supplement: Supplementary file 2 [file 41420_2026_3212_MOESM2_ESM.docx]

Supplementary Figure 1. Survival rate of mice with SAW established by CLP method.
